# Effects of Sodium-Glucose Cotransporter Inhibitor Use in Type 2 Diabetes Mellitus Patients With Heart Failure

**DOI:** 10.7759/cureus.34687

**Published:** 2023-02-06

**Authors:** Silpa Choday, Niriksha Ravi, Anusha Parisapogu, Blessing T Ojinna, Mingma L Sherpa

**Affiliations:** 1 Internal Medicine, California Institute of Behavioral Neurosciences & Psychology, Fairfield, USA; 2 Internal Medicine and Neurology, California Institute of Behavioral Neurosciences & Psychology, Fairfield, USA; 3 Infectious Diseases, Mayo Clinic, Rochester, USA; 4 Internal Medicine and Pediatrics, California Institute of Behavioral Neurosciences & Psychology, Fairfield, USA; 5 General Medicine, University of Nigeria Nsukka, College of Medicine, Enugu, NGA

**Keywords:** gliflozins, sglt2 inhibitors, high blood glucose, insulin resistance, diabetes mellitus, cardiac insufficiency, reduced ejection fraction, heart failure

## Abstract

The advances in the development of sodium-glucose cotransporter 2 inhibitors (SGLT2i) have expanded the variety of favorable approaches to treating diabetes mellitus. It is possible to have an improvement in insulin resistance and natriuresis by inhibiting the reabsorption of sodium and glucose at the proximal tubules in the kidney, and a decrease in cardiovascular mortality in patients with diabetes mellitus (DM). In addition, SGLT2i provides renoprotection by reducing intraglomerular higher blood pressure. The usage of SGLT2i also provides hemodynamic and metabolic benefits. SGLT2i demonstrates large cardiovascular benefits in patients both with and without diabetes, as well as in existing heart failure patients. These SGLT2i have direct and indirect effects on the kidney, likely contributing to stated cardiovascular benefits. Here we review the literature on the direct effects of SGLT2 inhibitors in diabetic patients with heart failure (HF). We assume that the benefit in cardiac cells modulated by SGLT2i is due to the inhibition of sodium transporters affecting intracellular sodium homeostasis. In conclusion, the sodium transporters in cardiac cells provide, at least partly, an example of the clinical benefits of SGLT2i observed in HF patients.

## Introduction and background

Type 2 diabetes mellitus (T2DM) is very common in the United States of America (USA). More than 30 million people in the USA live with T2DM and its prevalence is expected to rise every year. Diabetes is a leading cause of chronic kidney disease and end-stage renal disease (ESRD) which is observed to have a greater mortality risk [1]. T2DM is associated with a significant risk for renal and cardiovascular diseases including heart failure (HF) [2]. The complications of DM are related to microvascular and macrovascular disorders such as impaired renal function and volume overload. Patients with T2DM are at great risk of developing HF. In large-scale clinical trials, it has been shown that sodium-glucose 2 transport inhibitors (SGLTi) reduce the risk of HF events with existing cardiovascular disease or other risk factors, though it is not clear whether the benefits are observed among the broad spectrum of patients with HF; this includes cases of HF with preserved ejection fraction (HFpEF) and reduced ejection fraction (HFrEF) [2]. 

The introduction of sodium-glucose cotransporter 2 (SGLT2) inhibitors has shown a reduction in hospitalizations for HF patients in the CANVAS [3] and EMPA-REG OUTCOME trials [4]. Currently, a large-scale study is ongoing regarding SGLT2 inhibitors in chronic heart failure patients, and it is expected that they play a vital role in the management of HF [5]. 

The observation of increased HF rates with thiazolidinediones and their withdrawal from the market increased the trials of the new agents for cardiovascular safety, especially for HF patients [6]. Two new classes of drugs glucagon-like peptide 1 receptor agonists (GLP1Ra) and SGLT2 inhibitors might change the trajectory of lethal synergy for T2DM and HF. While both classes show comparable benefits on cardiovascular outcomes, their impact on HF events is different [7]. The effects of SGLT2i on cardiac cells target the pathologic mechanism of HF with cardiomyopathy [8]. This review summarizes the current knowledge of the direct effects of SGLT2 inhibitors on HF. 

## Review

SGLT2 inhibitors and diuretic activity

SGLT2 inhibitors have proven effective in reducing cardiac congestion without worsening kidney function in acute decompensated HF with reduced ejection fraction [8]. A reduction in cardiac preload has been noticed with the use of the SGLT2 inhibitor’s natriuretic effect. In the proximal tubule where is the reabsorption of sodium and glucose, they both are co-transported in the ratio of 1:1. With SGLT2 inhibitors, higher sodium reaches the distal tubule, causing an increase in sodium excretion, which in turn activates the renin-angiotensin-aldosterone system (RAAS) [9]. So, the reduction in plasma volume appears to be transient which can be detected at one week but not after 12 weeks [10]. A combination of SGLT2 inhibitors with a loop diuretic may result in reducing the interstitial volume compared with intravascular volume, thus resulting in less compensatory activation of RAAS [11]. It is suggested that the physicians monitor the volume status of the patients after initiation of SGLT2 inhibitors and decrease the dosing of loop diuretics in case dizziness or orthostatic hypotension is encountered [12].

SGLT inhibitors in heart failure with a reduced ejection fraction 

Recent guidelines 2022 released for the management of heart failure recommendations present an evidence-based approach to managing patients with heart failure, with the intent to improve the quality of care and align with patients’ interests [13]. The guidelines mentioned in the year 2022 recommend the use of SGLT2 cotransporters in the management of hyperglycemia and decrease the mortality and morbidity related to HF in patients who suffer from T2DM and HF [13] based on EMPEROR-Reduced [14], DAPA-HF [15], and DECLARE-TIMI 58 [16]. In other terms, they only reduce cardiovascular consequences in T2DM and heart failure with reduced ejection fraction. Dapagliflozin and canagliflozin improve surrogate endpoints in patients with HF and diabetes with a preserved ejection fraction of ≥ 45% [17,18]: "EMPEROR-Preserved released that empagliflozin reduces the combined risk of cardiovascular death or hospitalization for heart failure in patients with heart failure with preserved ejection fraction (more than 40%), regardless of the presence or absence of diabetes" [19].

SGLT inhibitors in HF with a preserved ejection fraction 

Gliflozins like empagliflozin and dapagliflozin have been shown to be effective in patients who experience symptomatic HF with reduced ejection fraction independent of T2DM [20]. Current therapies for patients with HF with preserved ejection fraction are limited and no drugs have proven effective yet. A meta-analysis-based study with limited data of VERTIS CV and DECLARE-TMI 58 trials has suggested some effects of SGLT2 inhibitors on HF hospitalizations in HF with preserved ejection fraction [21]. The study did not include a significant number of patients with HF with preserved ejection fraction in its inclusion criteria. The DELIVER trial studies the effects of dapagliflozin in patients with HF with preserved ejection fraction (with "EF >40%, structural heart disease, increased NT-pro BNP levels, NYHA II-IV") on cardiovascular death or HF events. Consequently, the EMPEROR-Preserved trial studies the effect of empagliflozin in patients with HF with preserved ejection fraction on cardiovascular death or hospitalization due to HF. Both clinical trials will be of greatest importance, as HF with preserved ejection fraction does not have enough evidence and unmet need in the field of cardiology [22].

SGLT2 inhibitors in HF

The efficacy of dapagliflozin in HF with reduced EF was first studied in the DAPA-HF trial which includes patients with T2DM and without T2DM in addition to worsening HF and cardiovascular death [23]. Dapagliflozin has been shown to reduce the outcome of worsening HF and death by 26%, but it caused an increase in all-cause mortality and reduced symptoms of HF symptoms which was assessed by the Kansas City Cardiomyopathy Questionnaire [23]. In another trial of EMPEROR study with HFrEF, the medication empagliflozin has been shown to reduce mortality from cardiovascular causes and hospitalization for HF [14]. In both trials, SGLT2i has significantly decreased unfavorable renal outcomes: "a recent meta-analysis combining DAPA-HF and EMPEROR-Reduced clearly indicated a consistent reduction of all-cause mortality in respective HF patients" [21].

Glomerular hyperfiltration in diabetes 

The kidney is one of the crucial organs for glucose homeostasis [24]. The proximal tubular cells contain sodium-glucose cotransporters, and they filter sodium and glucose and reabsorb 90% of the glucose [25]. SGLT2 expression increases up to 80% in diabetes mellitus. Diabetes can involve structural and functional modifications to the renal cells and tubules. The first phase in diabetic patients is glomerular hyperfiltration [26]. An excessive increase in the GFR downregulates the tubular glomerular feedback [27]. There is a significant increase in sodium reabsorption which is responsible for a rise in blood pressure [28]. The increase in reabsorption of glucose increases the activity of SGLT2 transporters which in turn worsens glycemic control and also promotes the loading of sodium which impairs blood pressure control [29]. One of the benefits of SGLT2i is they downregulate sodium glucose transporters and decrease the uptake of sodium, which lowers both the reabsorption of sodium and glucose. As less sodium is reabsorbed, there will be an improvement in the retention of fluid and also the status of increased glucose level [30].

## Conclusions

SGLT2 inhibitors have demonstrated improved outcomes in patients with cardiovascular and renal issues with type 2 diabetes mellitus. It reduces the rate of hospitalization for heart failure patients. Studies have shown to prioritize prescribing SGLT2i in patients with T2DM and have been recognized as a new therapy in patients with or without T2DM and with HFrEF. According to the guidelines, SGLT2i dapagliflozin has been recently approved for the treatment of HFrEF.
